# Computer aided selection of candidate vaccine antigens

**DOI:** 10.1186/1745-7580-6-S2-S1

**Published:** 2010-11-03

**Authors:** Darren R Flower, Isabel K Macdonald, Kamna Ramakrishnan, Matthew N Davies, Irini A Doytchinova

**Affiliations:** 1 School of Life and Health Sciences, University of Aston, Aston Triangle, Birmingham, B4 7ET, UK; 2 The Jenner Institute, University of Oxford, Compton, Berkshire, UK; 3 Current address: OncImmune Limited, Clinical Sciences Building, Nottingham City Hospital, Hucknall Rd. Nottingham, UK; 4 Current address: Medical Genetics Section, University of Edinburgh, Edinburgh, UK; 5 Institute of Psychiatry, King's College London, De Crespigny Park, London, UK; 6 Faculty of Pharmacy, Medical University of Sofia, 2 Dunav st., 1000 Sofia, Bulgaria

## Abstract

Immunoinformatics is an emergent branch of informatics science that long ago pullulated from the tree of knowledge that is bioinformatics. It is a discipline which applies informatic techniques to problems of the immune system. To a great extent, immunoinformatics is typified by epitope prediction methods. It has found disappointingly limited use in the design and discovery of new vaccines, which is an area where proper computational support is generally lacking. Most extant vaccines are not based around isolated epitopes but rather correspond to chemically-treated or attenuated whole pathogens or correspond to individual proteins extract from whole pathogens or correspond to complex carbohydrate. In this chapter we attempt to review what progress there has been in an as-yet-underexplored area of immunoinformatics: the computational discovery of whole protein antigens. The effective development of antigen prediction methods would significantly reduce the laboratory resource required to identify pathogenic proteins as candidate subunit vaccines. We begin our review by placing antigen prediction firmly into context, exploring the role of reverse vaccinology in the design and discovery of vaccines. We also highlight several competing yet ultimately complementary methodological approaches: sub-cellular location prediction, identifying antigens using sequence similarity, and the use of sophisticated statistical approaches for predicting the probability of antigen characteristics. We end by exploring how a systems immunomics approach to the prediction of immunogenicity would prove helpful in the prediction of antigens.

## Vaccines, vaccination, and vaccinology: a brief introductory orientation

Vaccines are agents – either molecular or supramolecular - which can stimulate protective immunity against microbial pathogens and the diseases they cause. Protective immunity is a specific and enhanced adaptive immune response to subsequent re-infection or, when luck holds, infection by related organisms. Such augmented immunity is mediated by the exacerbation of immune memory, which militates against the effects of infectious organisms. The word vaccine itself is derived from *vacca* (Latin for cow). [1][2][3].

It is a thing of near universal agreement that mass vaccination - synergising as it does with the herd immunity it helps engender - is the most effective and efficacious prophylactic treatment currently available for contagious disease. Humankind is commonly affected by over seventy infectious diseases, many of which are or will be targets for vaccines. There are in excess of fifty licensed vaccines, half of which are deemed to be in common use. Most vaccines prevent childhood infections or are used by travellers to tropical or subtropical regions; only a minority combat disease in third-world countries. As recently as the late 1960s, there were over 10 million cases of smallpox spread through 31 countries, with about two million deaths a year, yet now smallpox is wholly and totally eradicated. Poliomyelitis or Polio is the other key global disease close to eradication. In 1991, the Pan American Health Organization eliminated polio from the Western Hemisphere. In the First World, the annual death rate arising for contagious diseases such as polio, diphtheria, or measles is less than one in a thousand. The Global Polio Eradication Program has now greatly reduced the prevalence of Polio in the rest of the world. Only 784 cases of polio were reported in 2003. Nevertheless, Polio remains endemic in Nigeria, Afghanistan, Pakistan, and India.

Despite such outrageous and egregious success, many major issues persist. No licensed vaccines exist for HIV and malaria, two of the World Health Organization (WHO)’s three big global killers, and there are no realistic hopes for such vaccines appearing in the short to medium term. Bacille Calmette Guérin (BCG), the only vaccine licensed currently for the third major world disease, tuberculosis, has only limited efficacy [4]. Add to this the 35 new, previously unknown infectious diseases identified in the past 25 years: HIV, Marburg’s disease, SARS, dengue, West Nile, and over 190 human infections with the potentially pandemic H5N1 influenza. It is commonly believed that new contagious diseases will continue to emerge in the 21st century. The world of the 21st century is threatened by parasitic diseases and emerging zoonotic infections; antibiotic-resistant bacteria; and bioterrorism [2]

**Figure 1:**
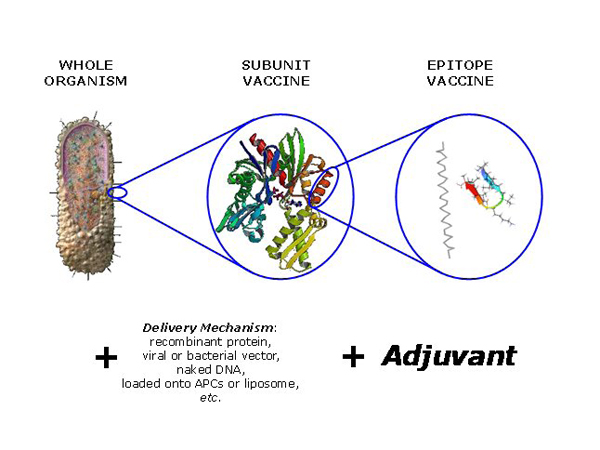
**A schema summarising the components of a generic vaccine.** The immunogenic component will typical be an attenuated or chemically-inactivated microbe, a protein antigen, or a poly-epitope or conjugate. This component will be combined with an appropriate delivery mechanism and an adjuvant. Delivery mechanisms and adjuvants overlap in their ability to increase the immunogenicity of a weakly-active vaccine.

The vaccine arena has long been neglected, partly as a consequence of the extraordinary success just adumbrated, but activity within it is now feverish and febrile [5,6]. Dozens of vaccine candidates have passed through phase II clinical trials, and during the past decade, vaccines in late development have numbered over 150. Unlike antibiotics, resistance to vaccines is negligible. In the same way that vaccines target many kinds of disease, themselves caused by microbial pathogens of all types, so there are many types of fundamentally distinct vaccine. See Figure 1. These include attenuated or inactivated whole pathogens, subunit vaccines, peptide vaccines, and vaccines based on carbohydrates, amongst others. Historically, the most successful and prevalent types vaccines have been those based on attenuated – that is “weaken” or non-infective - whole pathogen vaccines, for example BCG for TB or Sabin’s Polio vaccine. Safety concerns have fomented other vaccine strategies to develop, which focus on antigens and latterly epitopes as the intrinsic component of single or composite vaccines. Hepatitis B vaccine is an antigen - or subunit – based vaccine. While many epitope-based vaccines have now entered clinical trials, they are yet to fulfil their potential, medically or commercially.

## Antigens, immunogenicity, and subunit vaccines

Subunit vaccines are typically but not exclusively protein molecules and their discovery is often based around a somewhat haphazard, essentially empirical search for antigenic or immunogenic protein antigens. The word antigen has several meanings in immunology and thus in vaccinology. The Oxford English Dictionary defines an antigen as a “…foreign substance which, when injected into an organism, stimulates antibody production.” They trace its first use to 1908, and by the middle of the century the word had found its way into common technical dictionaries. An immunogen is on the other hand: “Any substance capable of eliciting an immune response”, although the word had other, earlier definitions. Its first use in its modern rendering is traced to 1959, but was doubtless in use before then.

Dictionary definitions seldom capture the scientific meaning of scientific terms particularly well. So, to be more precise, an immunogen, that is a moiety exhibiting immunogenicity, is any substance able to elicit a specific immune response, while an antigen - a moiety exhibiting antigenicity - is a substance which is recognized by an existing immune response, and its associated underlying molecular moieties such as T cells or antibodies, in a recall response. Immunogenicity is the most important and interesting property for vaccine design and discovery. It is this property that allows a molecular moiety to induce a significant immune response.

For the purposes of this review, our use of the terms antigen and immunogen shall be restricted to a single meaning; that of a protein, specifically one from a pathogenic microorganism, that evokes a measurable immune response. Currently, the prophylactic responses of almost all effective vaccines – with the exception of BCG which is mediated by the quadrille of interacting antigen presenting cells (APCs), T-cells, and other phagocytotic cells that characterise cellular immunity – are mediated by humoral immunity and antibodies. The pathogenesis of most diseases for which vaccines are not currently available are typically if not exclusively mediated by cellular immune mechanisms. Thus, when seeking to identify immune responses for untreated disease, the immunogenicity sought need not be mediated by antibodies; mediation by cellular mechanisms or a combination of humoral and cellular mechanisms is equally valid.

In what follows, we will endeavour to explore the availability of informatic tools and techniques for the identification of candidate subunit vaccines. Even today, in the early years of the 21^st^ century, many experimental scientists still retain an atavistic distrust of any and all computational approaches. The very suggestion that algorithms can help them, saving effort and time is an anathema to them and their way of thinking, undermining all that they hold dear. They are deeply distrustful of the reliability of computer methods, preferring what they perceive as the infallible reliability of the experiment. Not that they would admit to views so philosophically - and practically – naive; but deep down this is how they think and how they feel. Yet things have changed and changed dramatically, and things will continue so to change.

## Reverse vaccinology and epitopes

**Figure 2:**
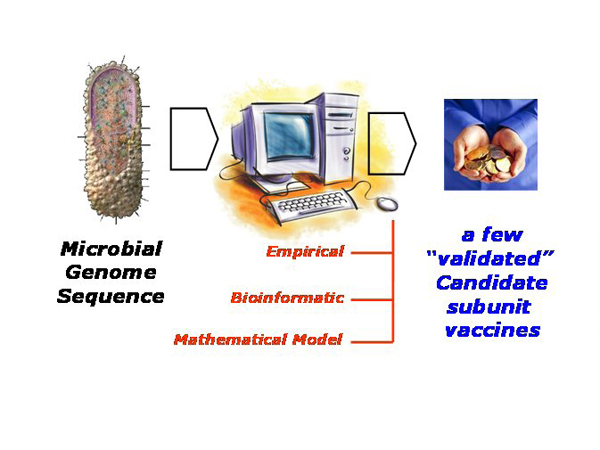
**Whole Antigen Discovery.** Taking a reverse vaccinology dynamic, the process of discovering candidate subunit vaccines begins with a microbial genome, perhaps newly sequence, progresses through an extensive computational stage, ultimately to deliver a shortlist of antigens which can be validated through subsequent laboratory examination. The computational stage can be empirical in nature; this is typified by the statistical approach embodied in vaxijen [98]. Or this stage can be bioinformatic; this involves predicting subcellular location and expression levels and the like. Or, this stage can take the form of a complex mathematical model which uses immunoinformatic models combined with mathematical methods, such as metabolic control theory [116], to predict cell-surface epitope populations.

Slowly but surely, reverse vaccinology is becoming a discernibly more prevalent means of identifying subunit vaccines; and slowly but surely the contribution made by computation to the practice of reverse vaccinology is becoming ever more significant and ever more obvious [7]. See Figure 2. Conventional laboratory-based, experimental microbiological approaches to antigen identification typically start with the cultivation of the target pathogenic micro-organism under laboratory conditions, then dissecting them into their component proteins, assaying these in some cascade of in vitro and in vivo assays, leading ultimately to the identification of proteins which display requisite protective immunity. It would indeed be wonderful if the identification of candidate vaccines was really and consistently this simple and systematic? Unfortunately, it is often so much more complex, confusing, and confounding. It is not always possible to cultivate a particular pathogen outside of the host organism. Many proteins are only expressed transiently during the course of infection. Nor are all proteins easily expressed in sufficient quantities *in vitro*. Thus many potential candidate vaccines may be missed.

Reverse vaccinology [7-10] is, by contrast, able to analyze a genome to identify potential antigens. Initially, the pathogenic genome is scanned for “open reading frames” or ORFs. Once all ORFs have been identified, proteins are selected on the basis that they will be accessible to immune system surveillance, usually using some form of informatic-based prediction methodology or, more likely, set of methdologies. Reverse vaccinology was established by a group studying Neisseria meningitidis, which is responsible for sepsis and meningococcal meningitis. Vaccines are available for all serotypes except subgroup B. Potential ORFs in *N. meningitidis* genome were identified [11][12]; 570 proteins were identified, 350 expressed in vitro, and 85 were surface exposed. Seven proteins conferred immunity over a broad range of strains. 

Another good example is Streptococcus pneumoniae, a major cause of sepsis, pneumonia, and meningitis [13][14]. 130 potential ORFs were identified, 108 of which could be expressed; six proteins were found to induce protection. Another example is Porphyromonas gingivalis, a gram-negative anaerobic bacterium found in subgingival plaques present in chronic adult inflammatory gum disease periodontitis. Of the 370 ORFS identified in the genome [15], 120 protein sequences were predicted to be open to surveillance by the immune system, 40 were positive for one or more sera. When used to vaccinate mice, two antigens exhibited clear protection.

This very brief survey highlights the potential for vaccine discovery using this approach. However, the number of potential antigens is still high, particularly when we explore all potential difficulties in expressing and characterising them. Both experimental and computational methodologies may thus omit potentially important antigenic proteins from analysis, albeit for different reasons, and both require continual optimisation. In particular, bioinformatics support for preclinical vaccine discovery has yet to flourish; but, as interest in vaccines waxes, this situation is changing: expanding from the nursery to at least the kindergarten. There are two main forms of support for vaccine discovery provided by bioinformatics. The first is technically indistinguishable from support for more general target discovery, and includes genomic annotation for both host and pathogen sequences and immunotranscriptomics, the application of microarray analysis to the immune system. The other type kind of support is focussed on immunoinformatics: it addresses problems such as the accurate prediction of epitopes.

Currently, prediction of T cell epitopes is essentially confined to predictions of varying accuracy of peptide binding to Major Histocompatibility Complex. Binding of peptides to class I MHC are reasonably accurate, at least for well characterised alleles [16]. However, several comparative studies have shown recently that the prediction of class II MHC binding prediction T-cell epitopes is typically poor [17][18][19], and likewise for structure-driven prediction of MHC-mediated epitopes [20][21]. Similarly, both structure- [22] and data-driven [23] prediction of antibody-mediated epitopes is sub-optimal. While the prediction of B cell epitopes remains primitive, or depends on an oft elusive knowledge of protein structure, methods for T cell epitopes prediction displaying not inconsiderable algorithmic sophistication have been and continue to be developed.

Despite this, and for different reasons, reliable and consistent prediction remains somewhat elusive; this regrettable decision will doubtless continue so for some time. Ultimately, all methods, however sophisticated, are severely circumscribed by the data used to propagate and parameterise them. No data-driven method can go beyond the data used to train it; all methods are likewise much superior in their ability to interpolate than their ability to extrapolate. It is only by compiling extensive, high-quality data that we can aspire to create excellent models of general applicability. What we require are sets of data that are complete, thorough, and properly thought through. Such sets would be able to explore both efficiently and effectively the complex and confounding multi-dimensional phase space of properties evinced by the molecules we target. 

IEDB [24-29] is a step-change in this direction. IEDB is the dominant force in current immunoinformatics and stands as one of the principal achievements of the field. The number and quality of epitopes within it are an enormous improvement over all that has gone before. Alex Sette and his co-workers are to be congratulated on their achievement, but, like all dominant things, IEDB has tended to choke most other efforts in the field, and even IEDB has its limitations. 

Data is usually multi-dimensional, and each dimension will typically be correlated, to a greater or lesser extent, with each other dimension. Together, these many dimensions chart a space: a space of structural variation or variation of properties. If the data we use is itself of sufficient quality and provides a good enough coverage of the space, then straightforward methods drawn from, say, computer science - of which there are indeed very many - are now of satisfactory accuracy to build models of high predictive accuracy.

As we have said, there is an on-going need for the quality, quantity, and availability of data to improve and increase. Prediction is enthral to its underlying data. Bias within the data places strict limitations upon the interpretability and generality of models from which it is derived. In general, for MHC-peptide binding experiments, the sequences of peptides studied are very biased in terms of amino acid composition, often favouring hydrophobic sequences. This arises, in part, from pre-selection processes that result in self-reinforcement. Binding motifs are often used to reduce the experimental burden of epitope discovery. Very sparse sequence patterns are matched and the corresponding subset of peptides tested, with an enormous reduction in sequence diversity.

Irrespective of their poor performance in prediction, several other problems exist, albeit different for T- and B-cell epitope prediction. For T-cell prediction, the key issue is the availability of data. It has recently been shown that T-cell epitopes, which were previously thought to be short peptides of 8-10 amino acids, can be up to 16 amino acids or perhaps even more. The existence of these longmer epitopes has greatly expanded the repertoire of peptides open to inspection by T-cells [30][31][32][33]. Problematic as this seems, the situation is made worse by the fundamental logistic constraints of sampling within the specificity exhibited by a single allele. The number of possible sequences that a nonameric peptide might possess is in the region of 512 billion; considering that a single model is built from at most a few hundred peptides, the sampling undertaken is infinitesimally small. Similarly, the 3000+ different MHC alleles known to exist in the global human population indicates the extraordinary potential for distinct peptide specificities within the potential patient cohort.

For B-cell prediction, explaining the poor performance of B-cell epitope prediction algorithms may point to a fundamental misinterpretation of extant epitope data. PEPSCAN is perhaps the most abundant data available currently but may not be what it seems. Experimentally derived epitopes are identified by being assayed against pre-existing antibodies with affinity for whole antigens. However, if such “epitopes” are mapped on to their parent antigen structure they are randomly located throughout the protein and do not equate to clear surface patches, as one might expect if they simply mimic discontinuous epitopes identified by crystallography; *in situ* these antigenic regions can be completely buried, and thus inaccessible to antibody binding, rather than exposed. If we compare the conformation of antibody-bound peptides with those from the intact antigens, they are usually quite different. However, B-cell epitopes in intact antigen and in whole antigen-antibody complexes are similar. It may be that the preformed antibody recognizes denatured antigen *in vivo* or that the isolated peptide adopts a conformation able to mimic the surface features of a discontinuous epitope.

Moreover, there is also evidence that the responsiveness of the immune system to pathogenic proteins is only poorly correlated with the possession of T cell epitopes, and that many potential epitopes have been deleted in proteins regularly accessible to immune surveillance, perhaps as an evolutionary counter measure in the war between host and pathogen [34]. Taken together, these factors suggest that methods which rely solely on the possession of epitopes are unlikely to be fully effective when tasked with identifying antigens or immunogens. This conjecture is confirmed by what information there is, which indicates that there is little simple correspondence between antigens selected on this basis and experimentally verified antigenic or protective proteins.

Thus we must seek alternative methods for selecting, and prioritising within that selection, proteins likely to be antigenic and protective. We shall below examine three key approaches: subcellular location prediction, sequence similarity, and empirical statistical approaches, typified by VaxiJen.

## Identifying antigens through subcellular location prediction

For a protein to be accessible to surveillance by the immune system, it is often assumed to be physically external to the microbial organism or at least present on its surface rather than being sequestered away far from the roving eye of the immune system. For bacteria this means it must be secreted or located on - or in - the outer membrane surface. In this context, immune surveillance is undertaken by a variety of cellular and molecular actors, but chiefly by neutralising antibodies. Thus the goal of identifying antigens through the use of sub-cellular location prediction is principally to identify surface proteins accessible to binding by neutralizing antibodies.

Unlike the relatively straightforward and well-studied task of identifying ORFs, selecting the subcellular location of proteins can be challenging to the point of confusion. Nonetheless, and notwithstanding the obvious caveats inherent in this strategy, this has become a favoured approach taken by many when tasked with selecting vaccine candidates.

We should remember, however, that antigens are not epitopes, and epitopes are not antigens. So possession of T-cell and B-cell epitopes is in certain senses independent of sub-cellular location, and they may in principle be possessed by any protein. As we have said, there is evidence for T-cell epitope depletion in pathogenic proteins [35]; or it may be that just picking immune-accessible proteins naturally favours proteins rich in antibody and helper T-cell epitopes, as opposed to cytotoxic T-cell epitopes, at least for membrane proteins. As data accumulates on the specific responses made to differently located accessible proteins we can build these recondite subtleties into prediction strategies.

There are two basic kinds of prediction method: manual construction of rules of what determines subcellular location and the application of data-driven machine learning methods, which determine factors that discriminate between proteins from different known locations. Accuracy differs markedly between different methods and different compartments, mostly due to a paucity of data. Data used to discriminate between compartments include the amino acid composition of the whole protein; sequence-derived features of the protein, such as hydrophobic regions; the presence of certain specific motifs; or a combination thereof.

Databases, such as ProDom [36], Pfam [37], and PROSITE [38], contain within them the capacity to identify sequence motifs characteristic of certain protein families; this can in turn help us to predict if a protein belongs to an family of proteins which are typically extracellular. However, gross sequence similarity alone is neither a necessary nor a sufficient condition for a protein to share a common sub-cellular location. Very similar sequences may be located quite differently, while lack of similarity is no guarantee that proteins will not be co-located. Moreover, many proteins can and do exist in several locations within the cell, and often evince different if related functions in these different locations [39]. Likewise, different organisms, and specifically eukaryotes versus prokaryotes, have quite different locations, both in number and in physical nature. The number of such compartments used in prediction studies varies considerably, and few papers address the full rich complexity of cell biology. For example, a common schema reduces eukaryotic cells to 4 compartments (cytoplasmic, extracellular, mitochondrial, and nuclear) and prokaryotic to 3 compartments (cytoplasmic, extracellular, and periplasmic), though some papers use over 10 compartments for eukaryotes. In fact, 10 compartments is an under-estimate, and the richness and the complexity of sub-cellular location is daunting, since any attempt to predict it must account for transient and permanent location, multiple locations, and both membrane organelles and multi-protein complexes as potential locales.

Among binary approaches, arguably the best method is SignalP, which employs neural networks and predicts N-terminal secretion signals cleaved by Signal Peptidase-I-and their associated cleavage site [40-42] . The signal predicted is the type II signal peptide common to both eukaryotic and prokaryotic organisms, for which there is a wealth of data, in terms of both quality and quantity. A recent enhancement to SignalP is the implementation of a hidden Markov model (HMM) which seeks to discriminate uncleaved signal anchors from cleaved signal peptides. One of the limitations of SignalP is overprediction, as it cannot reliably discriminate between several very similar yet distinct signal sequences, regularly predicting membrane proteins and lipopteins as type II signals. Many other kinds of signal sequence exist. A number of methods have been developed to predict lipoproteins, for example. The prediction of proteins that are translocated via the TAT-dependent pathway is also important but has yet to be addressed in any depth. 

Many programs and servers have been devised and developed able to predict the subcellular location of gene products and proteins. PSORT is a well-known, well-used example; it is knowledge-based, multicategory prediction method, composed of several programs [43-46]. PSORT I predicts 17 different subcellular compartments, while PSORT II predicts 10 locations. There are several specialized versions of PSORT. iPSORT deals specifically with secreted, mitochondrial, and chloroplast locations. PSORT-B only predicts bacterial sub-cellular locations. It reports precision values of 96.5% and recall values of 74.8%. Another effective program is HensBC [47]. This constructs a hierarchical ensemble of classifiers by applying a series of if–then rules. HensBC is able to assign proteins to one of four different types (cytoplasmic, mitochondrial, nuclear, or extracellular) with approximately 80% accuracy for gram-negative bacterial proteins. Another program is SubLoc [48], a client/server suite which offers an interface for the prediction of prokaryotic subcellular location in three compartments. Another still is Gpos-PLoc [49], which fuses many basic classifiers, engineered using the optimized evidence-theoretic K-nearest neighbours rule. Other programs include TatP 1.0 [50], LipoP 1.0 [51], and Phobius [52]. This list goes ever on and on. A comparison of a subset of these programs, which used a test set of 272 mycobacterial proteins [53], indicated that subcellular localization methods generally had high predictive specificity and recognised true negatives reliably.

We have also developed a range of programs specifically aimed at addressing the prediction of subcellular location within bacteria. Building on a set of algorithms able to predict membrane proteins, TAT secretion, and lipoproteins [54-60], a set of Bayesian networks, which were able to make individual predictions for specific subcellular locations was implemented in three pipelines with different architectures: a parallel implementation with a confidence level-based decision engine and two serial implementations with a hierarchical decision structure [61]. These two hierarchies were rooted differently, one by prediction between membrane types and the other by soluble versus membrane prediction. The parallel pipeline outperformed the serial pipeline, but took twice as long to execute. The soluble-rooted serial pipeline outperformed the membrane-rooted predictor. Assessment using genomic test sets was more equivocal, as many more predictions are made by the parallel pipeline, yet the serial pipeline identifies 22 more of the 74 proteins of known location.

This work is indicative of a clear direction that the development of future subcellular prediction strategies might take. Currently, the richness of subcellular structures is yet to be integrated into location prediction. Many cellular organelles are not included in such studies, nor are proteins with multiple locations, nor are the different routes by which proteins can reach the same compartment. The list of such caveats continues. Clearly, if we can develop high specificity predictors for each such compartment, then combining them appropriately [61], must be the way forward. There is then a need for a more sophisticated phase of supervised learning, where better and more complete annotation is built into any approaches taken. 

Many difficulties remain however, most notably the lack of validated, high quality datasets where the location of proteins has been established unambiguously. This paucity is particularly acute for certain important but less well-studied kinds of secreted protein, such as those secreted by the type III secretion system. While databases such as DBSubLoc [62] have been developed, their utility is open to question. So too is the extraction of datasets directly from SWISS-PROT. Here the inherent uncertainty in some of the available annotation can be confounding or at least frustrating. Likewise, much more work on the prediction of the subcellular location of viral proteins is required; while some studies have been undertaken [63][64], the complexity of their interactions with their host organism must be factored into future analyses. Generally, we can make the observation that ignoring this complexity is, in trying to simplify matters, more likely to render any analysis simplistic. Fortunately, subcellular location prediction, useful though it can be, and quibbles and cavils notwithstanding, is not the only method available to us. There are other weapons in our arsenal, many with an equal provenance and of equal utility.

## Identifying antigens through sequence similarity

One of the most obvious ways to identify new potential antigens in newly sequenced microbial genomes is through similarity searching. Assuming that we know the sequence of one or more extant antigens, we can make use sequence searching programs of various complexity and sophistication, such as BLAST or FASTA, to identify similar sequences in the target genome. This set of selected candidate antigens can then be prioritised for further theoretical and ultimately experimental validation. Obviously, all the same caveats that exist for any sequence search hold here also: which thresholds are appropriate? Are apparently high-scoring matches real or an artefact of search methodology? This process also presupposes that enough known antigens are available so that such searches can be comprehensive and thus effective. Such compilation is the role played by the database.

In the last decade, factory-scale experimentation allied to extensive literature mining has generated many functional immunology databases. Databases, such as SYFPEITHI [65,66], which focus on properties of cellular immunology, and look primarily at data relevant to MHC processing, presentation, and T-cell recognition have existed since the mid 1990s. Arguably, the best such database is the HIV Molecular Immunology Database [67], although clearly the depth of the database is at the expense of breadth and generality. It archives CD4+ and CD8+ T-cell and B-cell epitopes derived from the virus. It also includes detailed biological information regarding the response to the epitope, including its impact on long term survival, common escape mutations, whether an epitope is recognized in early infection, and curated alignments summarizing the epitope’s global variability.

Other recent databases include MHCBN [68,69], which contains 18,790 MHC-binding peptides, 3,227 MHC nonbinding peptides, 1,053 TAP binders and nonbinders, and 6,548 T-cell epitopes. EPIMHC [70] is a relational database of naturally occurring MHC-binding peptides and T-cell epitopes. Presently, the database includes 4867 distinct peptide sequences from various sources, including 84 tumor antigens. Two databases in particular, warrant special attention, albeit for different reasons. They are AntiJen [71], formerly known as Jenpep [72,73], and IEDB [24].

AntiJen seeks to integrate a wider range data than is archived by other databases. Implemented as a relational postgreSQL database, AntiJen is sourced from the primary literature and contains over 24,000 entries; it includes quantitative kinetic, thermodynamic, functional, and cellular data within the context of immunology and vaccinology. As well as T-cell and MHC binding data, AntiJen holds over 3,500 entries for linear and discontinuous B-cell epitopes, and includes measurements of peptide interactions with TAP transporter and peptide-MHC complex interactions with T-cell receptors (TCR), as well as immunological protein-protein interactions.

IEDB is a database lavishly funded by the NIH, which addresses issues of biodefence. As we have said, it is on a much larger scale than any other similar database, and benefits significantly from the input of 13 dedicated epitope sequencing projects. These exist, in part at least, to populate the database. IEDB has largely eclipsed other efforts in functional immune databases. However, it does not, as a priority, address antigenicity at the whole protein level.

At this point it is worth discussing the distinction between functional annotation and the objective discussed here. Generally speaking, the function that a protein performs within the context of its organism of origin is irrelevant to its status as an antigen. Here the ubiquity and multiple meanings of the word antigen are of little if any help. A protective antigen is a protein which is recognised and recalled by the host. This characteristic does not seem to be linked to the fact that a protein is an enzyme or a DNA binding protein, nor does logic require such a link. Thus identifying function is not a necessary condition for a protein to be an antigen, though the unequivocal identification of certain functions, such as being a virulence factor for example, greatly increases the probability that it will be such.  

Concerning antigens, however, these databases, although replete with information concerning individual B cell epitopes, T cell epitopes, and Major Histocompatibility Complex (MHC) binding peptides, remain otherwise partial and incomplete. Their focus is on the epitope, not the antigen. There are many antigens for which specific epitope or MHC binding information is not currently available, yet many such antigens are known experimentally to induce either or both innate or adaptive immune responses. Fortunately for the future of vaccine design and discovery specific antigen-orientated – rather than epitope-orientated - databases are now becoming available.

Arguably, the clearest and most unequivocal example of an antigenic protein is the so-called virulence factor (VF). These proteins are able to undertake the colonization of a host organism and/or induce disease. They are the front-line weapons in the pathogenic arsenal. Analysis of known pathogenic species, such as Vibrio cholerae or Streptococcus pyogenes, has enabled the recognition of recurrent “systems” of VFs and toxins that may total 40 or more distinct proteins. These may exist as discrete pathogenicity islands or be spread more widely in the genome.

Traditionally, classification of VFs has categorised them as belonging to several thematic groups: adherence/colonization factors, invasions, exotoxins, transporters, iron-binding siderophores, and miscellaneous cell surface factors. A broader definition groups VFs into three classes: (1) “true” virulence factors; (2) VFs associated with the expression and regulation of class 1 VF genes; and (3) VFs required for the colonization of the host [74].

A number of databases that archive VFs have been reported. The Virulence Factor Database (VFDB; URL: http://www.mgc.ac.cn/VFs/) contains 16 characterized bacterial genomes with an emphasis on functional and structural biology and can be searched using text, BLAST, or functional queries [75,76]. TVFac (Los Alamos National Laboratory Toxin and Virulence Factor database; URL: http://www.tvfac.lanl.gov/) contains genetic information on over 250 organisms and separate records for thousands of virulence genes and associated factors. The Fish Pathogen Database (URL: http://www.fishpathogens.eu/vhsv/index.php), set up by the Bacteriology and Fish Diseases Laboratory, has identified over 500 virulence genes using fish as a model system. Pathogens studied include Aeromonas hydrophila, Edwardsiella tarda, and many Vibrio species. Candida albicans virulence factor (CandiVF) is a small species-specific database that contains VFs, which may be searched using BLAST or a HLA-DR hotspot prediction server [77]. PHI-base is a noteworthy development as it integrates VFs into a cohesive whole a variety of pathogens of plants and animals [78].

Obviously, antigens need not be VFs, they need only be accessible to immune surveillance and need not be directly or indirectly involved in infectivity. Because of this, other types of database are required, able to capture and contain a wider tranche of relevant data. In the recent past, another database has been developed: AntigenDB [79] contains a compilation of over 500 antigens drawn from the literature and other immunological resources. It marks a new beginning in immunoinformatics, signalling a switch away from the peptide epitope and toward the whole protein antigen. These antigens come from 44 important pathogenic species. In AntigenDB, a database entry contains information regarding the sequence, structure, origin, etc. of an antigen with additional information such as B and T-cell epitopes, MHC binding, function, gene-expression and post translational modifications, where available. AntigenDB also provides links to major internal and external databases. AntigenDB will be updated on a rolling basis, with the regular addition of antigens from other organisms. This database will form the core of future attempts to predict antigens both by sequence similarity and using more recondite analysis.

At this point it is worth mentioning the issue of thresholds. Clearly, when one runs a sequence search, using BLAST for example, one might generate huge lists of near-identical proteins or get no hits at all; and, for that matter, we could also obtain almost any intermediate result. The issue is to judge which result is useful and which is not. This typically equates to setting a threshold, above which we anticipate usefulness and below which we might expect a lack of utility. Setting such thresholds is however no easy task. They are dependent on the nature of the family in question. For the lipocalin family [80-82], for example, hits are still valid in terms of structure and function at levels that would simply be noise for most other protein families. Thus thresholds are family dependent, as well as problem dependent. Empirically-selected cut-offs are thus the order of the day, but much thought and experimentation is needed in order to select appropriate values.

As well as seeking similarity to known antigens, there is another, quite prevalent, idea that is deserving of comment: that the immunogenicity of protein is determined solely by its lack of similarity to the host. What we search for is some quantitatively-meaningful measure of the “foreignness” of a protein which correlates highly with its immanent immunogenicity. In this context, what we mean by the word “foreignness” is the evolutionary distance between the host – a man or a cow or mouse - and the candidate antigen, or the organism that produced it, or both. Some consider this to be the prime factor determining the potential immunogenicity of a protein [83,84]. Clearly, since we are dealing with proteins and carbohydrates and the like, this evolutionary distance must be specified in terms of their molecular structures, or more likely their sequences, rather than in terms of morphological features.

The potential importance of such a concept is supported by the observation that immune systems are actively educated to lack reactivity when presented with self-proteins [85], a process – often called immune tolerance – which is generated via epitope-specific mechanisms including clonal anergy, receptor editing, and clonal deletion [86][87]. But how can we progress beyond this rather inexactly-specified philosophical standpoint to something which is practically useful when we select vaccine candidates?

A potentially more useful way to express this conjecture is that the likelihood that a protein is immunogenic is solely a function of that protein’s dissimilarity to the whole host proteome at the sequence level. Or, to be more precise, how close in terms of sequence similarity is the candidate to the closest or most similar member of the host proteome. Most sequence software is well suited to solving this problem, since it is precisely this problem that they were written to address. More difficult is assessing average dissimilarity to the whole proteome, a problem compounded when we use the similarity of overlapping peptide fragments rather than looking at the similarity at the level of global sequence alignment. In terms of choosing the length of such fragments, the epitope would seem to be the most logical choice, since this immunological quantum is the moiety most likely to be recognised during the immune response.

Yet even at the level of the epitope, a peptide of say 10 amino acids, even one mismatch in an otherwise perfect match can be significant, since such sequence differences, comprising a single amino acid, may exacerbate or abrogate neutralizing antibodies binding to a particular antigenic protein. Moreover, the cross-reactivity of a single high-affinity monoclonal antibody is rather different in nature to the cross-reactivity of large set of less affine polyclonal antibodies, and so too their ability to tolerate amino acid mismatches. It will also vary between individuals, since the immunization history of each organism will dictate to a large extent the recognition of epitopes.

Our understanding of epitopes can inform our understanding of mismatch tolerance, since the affinity of T-cell epitopes is more dependent on the possession of suitable anchor residues than it is on the possession of non-anchor residues. Having said, the dogma of anchor-dependent affinity was long ago debunked, since all residues make some kind of contribute to affinity, entropic or enthalpic, although generally it is right to say that so-called anchors do make more significant contributions. Our understanding of the structural-basis that determines the affinity of antibody-mediated epitopes is much less assured and complete, and the underlying thermodynamic causes of affinity, if strict causes they are, typically only become clear when high resolution structural data combines with measured thermodynamic metrics.  

Likewise, when one looks not at a representative individual, but at the whole population, then the deletion of a single protein, within one host versus another, can render candidates previously valid and immunogenic as suddenly neither. Again, these are difficult issues; as yet, they remain unresolved.

 Given the hypothesis that immunogenicity is in some sense mediated by the level of similarity between a pathogenic protein and the host proteome, we have, in as yet-unpublished work, sought to bench-mark sequence similarity analysis as a means of quantifying the differences between populations of antigens and non-antigen. To that end, we identified sets of 100 known antigenic and 100 non-antigenic protein sequences derived from a variety of sources: allergens, bacteria, fungi, parasites, tumours and viruses. These were compared to the human and mouse genomes using standard sequence similarity searching protocols. Whole pathogen proteomes were also aligned to these host proteomes. Most antigenic and non-antigenic sequences were observed to be non-redundant; this implies a lack of clear homologues between pathogens and the human or mouse proteomes, although a number of parasite antigens were found to have a much higher level of similarity. These proteins comprised heat shock proteins, catalases, and enzymes involved in hydrolysis. These protein families are structurally conserved, though they might display significant functionality diversity.

We also used statistical approaches such as the non-parametric Mann-Whitney test to assess if comparisons between the two populations were significant. The statistical null hypothesis was accepted in most cases, signifying that the effect presumably resulted solely from chance. The Mann-Whitney test supported the observations from sequence similarity analysis. We were unable to determine a threshold or cut-off based on the hypothesis of non-redundancy to the host’s proteome. These results suggest that this is not in itself a solution to the identification of antigens. A variant based on fragments may be more successful, and this is clearly an issue crying out for further, deeper research.

There are, of course, many other ways to approaches identifying antigenic proteins. One notable way, is looking for the horizontal transfer of so-called pathogenicity islands, clusters of pathogenic proteins acquired by transfer between micro-organisms. Detection of such islands, which are typically large gene clusters with an atypical yet characteristic G + C content, can in turn lead to the identification of antigenic proteins [88-91]. Analysis at the nucleic acid level rather than at the protein level can facilitate the discovery of virulence proteins, perhaps using similar approaches to that used to identify CpG islands [92][93][94][95].

However, rather than look at nucleic acid sequences, or at protein sequences directly, a new approach, based upon alignment-free techniques, has been developed which shows significant potential; we examine this next. 

## Identifying antigens through empirical statistical methods

 Most *in silico* approaches to predicting antigens make use of bioinformatics tools of one kind or another. Such tools can identify membrane proteins, signal peptides, or lipoproteins with some success, yet most algorithms still rely on sequence alignment to identify characteristic sequence relationships or motifs characteristic of antigens. This may prove problematic in several ways. Some proteins formed through divergent or convergent evolution lack obvious sequence similarity, although they may share similar structures and/or biological properties [96,97]. In such a situation, alignment-based approaches may produce ambiguous results or fail spectacularly.

Many methods presuppose a direct sequence relationship such as that which can be revealed by simple sequence search techniques, such as BLAST. This is not always the case. Immunogenicity, as a property, may instead be encoded within the sequence and structure of a protein in such a cryptic and subtle manner as to go beyond the conventional limitations imposed when one seeks direct identification by sequence alignment protocols. Likewise, the discovery of truly novel antigens will be frustrated by their lack of similarity to antigens of known provenance.

As a departure from alignment-dependent techniques, which dominate immunoinformatics as much as they do bioinformatics, we have implemented an approach which seeks to discriminate between candidate vaccines and nonantigens, using an alignment-free sequence representation [98,99]. Rather than concentrate on epitope and nonepitope regions, the method uses data on immunoprotective antigens derived from pathogenic sources to derive statistical models for predicting whole-protein antigenicity. In attempting to overcome the limitations imposed by alignment-dependent sequence similarity methods, we have implemented a novel alignment-independent method for antigen identification based on auto cross covariance (ACC) transformation of protein sequences into uniform equal-length vectors. The ACC transform is a protein sequence mining method originally devised by Wold *et al*. [100,101], which has found much application to the quantitative structure-activity relationships (QSAR) study of peptides and also for the classification of proteins [102-109]. The principal properties of the amino acids were represented by *z* descriptors [110][111][112], which describe amino acid hydrophobicity, molecular size and polarity. The ACC transformation accounts for neighbour effects, i.e. the lack of independence between different sequence positions. 

 Initially, we sought to develop models able to distinguish immunoprotective proteins from the general microbial proteome. We applied ACC pre-processing to sets of known bacterial, viral and tumour antigens and developed alignment-independent models for identifying antigens. In a separate paper, extra models were added which address fungal and parasite antigens. For bacterial, viral and tumour antigens, models had prediction accuracies in the 70% to 89% range [98,99][113]. For the parasite and fungal antigens, models had good predictive ability with 78% to 97% accuracy under internal cross validation in 7 groups. Under external validation, they gave 69% sensitivity ranking the true immunoprotective proteins in the first 25% of their proteomes.

The models were implemented in a server for the prediction of protective antigens and subunit vaccines, which we have christened VaxiJen [98] (URL: http://www.darrenflower.info/VaxiJen). The accuracy values noted above are indicative of an approach that is more accurate than has been seen before; for example, for B-cell epitope prediction. VaxiJen is an imperfect beginning; future research will yield significantly more insight as the number of known protective antigens increases [79].

## Antigen selection and immunogenicity

There are several bioinformatics problems unique to immunology: the foremost and greatest is the challenge of accurately predicting immunogenicity. The successful computational prediction of immunogenicity may manifest itself at one extreme in the identification of a T-cell or B-cell epitope. This is mediated by straightforward if not uncomplicated molecular recognition events, which can be understood through properly exploring relationships between fundamental physical properties – hydrogen bonding and lipophilicity for example – and apparent biological activity. Establishing such structure-activity or property-activity relationships is a considerable concern of current immunoinformatics. At the other extreme, we encounter an altogether more complex phenomenon, where we see immunogenicity made manifest in the accurate estimation of antigenicity at the whole protein level. This is a system property; that is a property of the whole immune system rather than an individual molecular recognition event. At present, this is very much a secondary aspect of modern day immunoinformatics.

The task of predicting whole protein immunogenicity is probably several orders of magnitude more complex and demanding than say predicting the binding of peptides to an MHC or even of predicting the binding of a pMHC complex to a TCR. This is not to say that such calculations are in any way trivial or lacking difficulty, but rather than that such complex prediction exercises are themselves subsumed in the task of predicting immunogencity at the system level.

**Figure 3:**
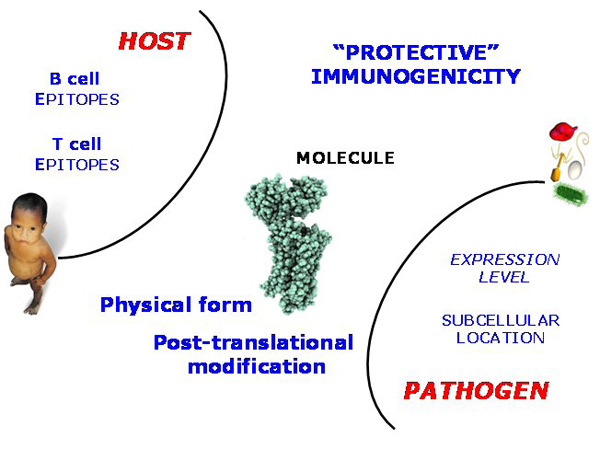
**Factors underlying Immunogenicity** As elaborated in the text, the phenomenon of Immunogenicity can be explored through the diversity of underlying individual factors contributing to the instigation of the immune response. These factors can be assigned to the host (epitope recognition), the pathogen (location and expression level), and also factors intrinsic to the protein antigen itself, such as the possession of post-translational danger signals.

In our view, and in the view of other commentators, the clinical manifestation of the immunogenicity of a vaccine antigen, as opposed to the immunogenicity of an isolated epitope, arises as the very complex amalgam of many intertwining intrinsic and extrinsic factors, operating at various length-scales and at various different rates. Such factors include host-side properties and pathogen-side properties, as well as protein-side properties that arise more or less exclusively from the protein itself. See Figure 3.

So-called host-side properties are properties intrinsic to the immune system of the host. They include the possession of B-cell epitopes or T-cell epitopes, as recognised by the adaptive immune system, or the possession of Pathogen-associated Molecular Patterns or PAMPs, which are recognised by Pattern Recognition Receptors, such as Toll-like receptors (TLRs), part of the innate immune system. Pathogen-side properties are properties intrinsic to the pathogen as a whole organism. They include the expression level evinced by an antigen, the time-course of its expression and secretion, and also its subcellular location. Protein-side properties include the state of aggregation exhibited by a candidate vaccine and any post-translational danger signals that the protein might possess. Some of these properties have been discussed before. Thus one would expect, at least naively, that a *bona fide* vaccine antigen would be both highly expressed and available for immune surveillance, as well as possessing epitopes that the host can recognise, where as a protein which is not immunogenic would lack such characteristics.

Identifying and predicting this diverse tranche of properties is a problem and thus a challenge. Indeed, each component part is a challenge unto itself. Consider the prediction of a T cell epitope: the best understood and most accurate of immunoinformatic prediction problems. Such an epitope needs to be processed properly into a population of different peptides, and for the processed peptides to exist in a reasonable amount, and then be bound by an MHC with reasonable affinity, before being recognized appropriately within the context of a pMHC complex by a TCR. This then is a complex and contingent process, similar to a generic Markov process, comprised of many stages which are themselves dependent on preceding steps. In terms of both mechanism and what steps-within-steps there may be, many of these steps are less than adequately understood. However, each step remains amenable to statistical evaluation. All stages are inherently predictable given an appropriate accumulation of relevant data.

The situation is very similar but not identical for biopharmaceuticals. In this case, we can dispense with the pathogen and replace it with several classes of extrinsic factors which include amongst others product manufacture, patient health, and medication strategy. Acute illness, particularly systemic illness, or latent bacterial or viral infections such HCV, can have a most profound effect on the manifestation of immunogenicity. Likewise, a patient’s genetics may alter profoundly the immune reaction to biopharmaceuticals, most obviously, the MHC haplotype will affect host T-cell responses.

The NERVE program is an expert system for vaccine antigen discovery, which addresses in a practical way some of the issues of prioritisation discussed above. The program has been developed to help automate the process of reverse vaccinology [114]. The identification and prioritising of potential ORFs using NERVE comprises 6 stages. Firstly, the prediction of subcellular localization; then whether the protein is an adhesin; followed by the identification of transmembrane domains; then the protein is compared with both the human and pathogen proteomes; after which the protein is finally assigned a suggestive function. Vaccine candidates are thus filtered and ranked, and a list of proteins is produced graded by precedence and the probability that it will be an immunogenic antigen.

But NERVE and other extant programs, such as DyNAVacS [115], tasked with such a confounding task necessarily fall far short of what is needed or indeed what is possible. There needs to be a concerted and far-reaching effort to address this issue. The most direct line of attack is to address first the subcellular location issue, as we discussed above. Likewise, we can deconstruct the antigen presentation pathway, building models for each step and then integrating them into a fully functional model. We can develop empirically-based, statistically-grounded approaches - of which VaxiJen [98,99,113] is merely the vanguard – that have their basis in emerging antigen databases. Yet all of this is just the beginning. We need to factor in B-cell and antibody mediated issues using structural data. We need to properly assess the role of aggregation, of expression levels, of post-translational danger signals and the possession of pathogen-derived PAMPs, as well as the ability of large molecule and small molecule adjuvants to raise the intrinsic immunogenicity of candidates to useful levels. We should note in passing that many host- derived molecules are also labelled PAMPs; these include fibronectin, HSP70, and heparan sulphate, amongst others. However, these molecules are endogenous activators of innate immunity and not relevant to this discussion, though they may figure in auto-immune scenarios. 

## Conclusions

Vaccines are a good thing; putting caveats and cavils to one side, no one sensible really doubts the truth of this statement. Vaccines have proved their worth time and again, but for them to continue to prove their worth we must find new ways of making them. Almost all present-day vaccines are mediated by antibodies, and the majority target viral diseases. Unfortunately, we are now running short of target diseases which fit this restrictive bill. Many of the pathogens responsible for current recalcitrant and emergent diseases are, and are set to prove, much more demanding and difficult to target. In many senses, the low hanging fruit has been cut down and now the fruit we most desire is well out of reach. Many diseases, including the WHO’s big three diseases: HIV, TB, and Malaria, are much more complicated functionally and/or structurally, and cellular, as opposed to humoral, immunology is tasked with defence against these dark arts.

Peptide vaccines and those based on APCs are important new if as yet unspectacular directions for research in vaccine discovery, yet modern strategies for vaccine development hinge primarily upon the effective search for vaccine antigens, at least for the proactive search for vaccines targeting long-standing but untreated diseases rather than the reactive response to so-called pandemics. Such antigens, once discovered, will, in time, and with careful manipulation and an appropriate delivery system and/or appropriate adjuvant, become first candidate subunit vaccines and, after proper clinical evaluation, the vaccines of tomorrow. There are, as we have outlined, many competing ideas, thoughts, and concepts that can help us in our quest. Certain of these hypothesis we have had cause to outline, are indubitably persuasive, even compellingly convincing, yet in execution many such methods fall far short of our desires. No single approach, however promising, is able to deliver on its promise.

The reason for this failure is comparatively simple if uncompromisingly unsatisfying; as we are dealing with over simplified abstractions, we cannot hope to capture what is necessary for prediction by looking in a relatively superficial way at a single contributory factor, since protein immunogenicity arises from many, many factors. This is not a bioinformatics problem that is easily solved; instead it is like protein secondary structure prediction which has resisted all attempts over many decades, so obscure and recondite and far removed from direct experience a problem as it is. Factors mediating protein immunogenicity are many and include host-side properties - possession of B or T cell epitopes for example - and pathogen-side properties - protein expression levels and sub-cellular location - as well as its aggregation state and the possession of post-translational danger signals. A candidate vaccine should be highly expressed, available for immune surveillance, and possess epitopes that the host recognises. Predicting such diverse properties remains challenging, though several contributing factors can be reliably predicted.

Yet, no one factor is itself enough. What we need is an integrative, systems biology approach to the problem. As the poet Maya Angelou so neatly puts it: “We all should know that diversity makes for a rich tapestry, and we must understand that all the threads of the tapestry are equal in value no matter what their colour.” No one method is universally applicable and successful; rather we need to integrate several equally-valid, equally-partial methods and draw from their synergy, useful, helpful data. This is the as yet unattained goal; yet as these are issues of such importance even a partial solution would be prize enough.

## Abbreviations

ACC: Auto Cross Covariance; APC: Antigen Presenting Cell; BCG: Bacille Calmette; Guérin; BLAST: Basic Local Alignment Search Tool; IEDB: Immune Epitope Database; HIV: Human Immunodeficiency Virus; MHC: Major Histocompatibility Complex; NIH: National Institutes For Health; ORF: Open Reading Frame; PAMP: Pathogen-Associated Molecular Patterns; PRR: Pattern Recognition Receptors; SARS: Severe Acute Respiratory Syndrome; TAP: Transporter Associated With Presentation; TB: Tuberculosis; TCR: T-Cell Receptor; TLR: Toll-Like Receptors; Tvfac: Toxin And Virulence Factor Database; VF: Virulence Factor; VFDB: Virulence Factor Database; WHO: World Health Organisation; Candivf: Candida Albicans Virulence Factor.

## Competing interests

The authors declare no competing financial interests.

## Author contributions

DF wrote the paper. IM wrote the paper. KR wrote the paper. MD wrote the paper. ID wrote the paper.

## References

[B1] Vivona S Gardy JL Ramachandran S Brinkman FSL Raghava GPS Flower DR Filippini F Computer-aided biotechnology: from immuno-informatics to reverse vaccinology. Trends Biotechnol 2008 26 190 200 10.1016/j.tibtech.2007.12.006 18291542

[B2] Flower D Bioinformatics for Vaccinology 2008 1 Wiley

[B3] Flower DR Davies MN Ranganathan S Bioinformatics for Immunomics 2010 1 Springer

[B4] Lambert PH Hawkridge T Hanekom WA New vaccines against tuberculosis. Clin Chest Med 2009 30 811 826 x 10.1016/j.ccm.2009.08.014 19925969

[B5] Vivona S Gardy JL Ramachandran S Brinkman FS Raghava GP Flower DR Filippini F Computer-aided biotechnology: from immuno-informatics to reverse vaccinology. Trends Biotechnol 2008 26 190 200 10.1016/j.tibtech.2007.12.006 18291542

[B6] Davies MN Flower DR Harnessing bioinformatics to discover new vaccines. Drug Discov Today 2007 12 389 395 10.1016/j.drudis.2007.03.010 17467575

[B7] Bambini S Rappuoli R The use of genomics in microbial vaccine development. Drug Discov Today 2009 14 252 260 10.1016/j.drudis.2008.12.007 19150507 PMC7108364

[B8] Serruto D Rappuoli R Post-genomic vaccine development. Febs Lett 2006 580 2985 2992 10.1016/j.febslet.2006.04.084 16716781

[B9] Mora M Donati C Medini D Covacci A Rappuoli R Microbial genomes and vaccine design: refinements to the classical reverse vaccinology approach. Curr Opin Microbiol 2006 9 532 536 10.1016/j.mib.2006.07.003 16890009

[B10] Serruto D Adu-Bobie J Capecchi B Rappuoli R Pizza M Masignani V Biotechnology and vaccines: application of functional genomics to Neisseria meningitidis and other bacterial pathogens. J Biotechnol 2004 113 15 32 10.1016/j.jbiotec.2004.03.024 15380644

[B11] Tettelin H Saunders NJ Heidelberg J Jeffries AC Nelson KE Eisen JA Ketchum KA Hood DW Peden JF Dodson RJ Complete genome sequence of Neisseria meningitidis serogroup B strain MC58. Science 2000 287 1809 1815 10.1126/science.287.5459.1809 10710307

[B12] Pizza M Scarlato V Masignani V Giuliani MM Arico B Comanducci M Jennings GT Baldi L Bartolini E Capecchi B Identification of vaccine candidates against serogroup B meningococcus by whole-genome sequencing. Science 2000 287 1816 1820 10.1126/science.287.5459.1816 10710308

[B13] Wizemann TM Heinrichs JH Adamou JE Erwin AL Kunsch C Choi GH Barash SC Rosen CA Masure HR Tuomanen E Use of a whole genome approach to identify vaccine molecules affording protection against Streptococcus pneumoniae infection. Infect Immun 2001 69 1593 1598 10.1128/IAI.69.3.1593-1598.2001 11179332 PMC98061

[B14] Maione D Margarit I Rinaudo CD Masignani V Mora M Scarselli M Tettelin H Brettoni C Iacobini ET Rosini R Identification of a universal Group B streptococcus vaccine by multiple genome screen. Science 2005 309 148 150 10.1126/science.1109869 15994562 PMC1351092

[B15] Ross BC Czajkowski L Hocking D Margetts M Webb E Rothel L Patterson M Agius C Camuglia S Reynolds E Identification of vaccine candidate antigens from a genomic analysis of Porphyromonas gingivalis. Vaccine 2001 19 4135 4142 10.1016/S0264-410X(01)00173-6 11457538

[B16] Lafuente EM Reche PA Prediction of MHC-peptide binding: a systematic and comprehensive overview. Curr Pharm Des 2009 15 3209 3220 10.2174/138161209789105162 19860671

[B17] Gowthaman U Agrewala JN In silico tools for predicting peptides binding to HLA-class II molecules: more confusion than conclusion. J Proteome Res 2008 7 154 163 10.1021/pr070527b 18034454

[B18] El-Manzalawy Y Dobbs D Honavar V On evaluating MHC-II binding peptide prediction methods. Plos One 2008 3 e3268 10.1371/journal.pone.0003268 18813344 PMC2533399

[B19] Lin HH Zhang GL Tongchusak S Reinherz EL Brusic V Evaluation of MHC-II peptide binding prediction servers: applications for vaccine research. Bmc Bioinformatics 2008 9 Suppl 12 S22 10.1186/1471-2105-9-S12-S22 19091022 PMC2638162

[B20] Knapp B Omasits U Frantal S Schreiner W A critical cross-validation of high throughput structural binding prediction methods for pMHC. J Comput Aided Mol Des 2009 23 301 307 10.1007/s10822-009-9259-2 19194661

[B21] Zhang H Wang P Papangelopoulos N Xu Y Sette A Bourne PE Lund O Ponomarenko J Nielsen M Peters B Limitations of Ab initio predictions of peptide binding to MHC class II molecules. Plos One 2010 5 e9272 10.1371/journal.pone.0009272 20174654 PMC2822856

[B22] Ponomarenko JV Bourne PE Antibody-protein interactions: benchmark datasets and prediction tools evaluation. Bmc Struct Biol 2007 7 64 10.1186/1472-6807-7-64 17910770 PMC2174481

[B23] Blythe MJ Flower DR Benchmarking B cell epitope prediction: underperformance of existing methods. Protein Sci 2005 14 246 248 10.1110/ps.041059505 15576553 PMC2253337

[B24] Vita R Zarebski L Greenbaum JA Emami H Hoof I Salimi N Damle R Sette A Peters B The immune epitope database 2.0. Nucleic Acids Res 2010 38 D854 862 10.1093/nar/gkp1004 19906713 PMC2808938

[B25] Vita R Peters B Sette A The curation guidelines of the immune epitope database and analysis resource. Cytometry A 2008 73 1066 1070 18688821 10.1002/cyto.a.20585 PMC2597159

[B26] Zhang Q Wang P Kim Y Haste-Andersen P Beaver J Bourne PE Bui HH Buus S Frankild S Greenbaum J Immune epitope database analysis resource (IEDB-AR). Nucleic Acids Res 2008 36 W513 518 10.1093/nar/gkn254 18515843 PMC2447801

[B27] Sette A The immune epitope database and analysis resource: from vision to blueprint. Genome Inform 2004 15 299 16312048

[B28] Peters B Sidney J Bourne P Bui HH Buus S Doh G Fleri W Kronenberg M Kubo R Lund O The design and implementation of the immune epitope database and analysis resource. Immunogenetics 2005 57 326 336 10.1007/s00251-005-0803-5 15895191 PMC4780685

[B29] Peters B Sidney J Bourne P Bui HH Buus S Doh G Fleri W Kronenberg M Kubo R Lund O The immune epitope database and analysis resource: from vision to blueprint. PLoS Biol 2005 3 e91 10.1371/journal.pbio.0030091 15760272 PMC1065705

[B30] Tynan FE Burrows SR Buckle AM Clements CS Borg NA Miles JJ Beddoe T Whisstock JC Wilce MC Silins SL T cell receptor recognition of a 'super-bulged' major histocompatibility complex class I-bound peptide. Nat Immunol 2005 6 1114 1122 10.1038/ni1257 16186824

[B31] Tynan FE Borg NA Miles JJ Beddoe T El-Hassen D Silins SL van Zuylen WJ Purcell AW Kjer-Nielsen L McCluskey J High resolution structures of highly bulged viral epitopes bound to major histocompatibility complex class I. Implications for T-cell receptor engagement and T-cell immunodominance. J Biol Chem 2005 280 23900 23909 10.1074/jbc.M503060200 15849183

[B32] Burrows SR Rossjohn J McCluskey J Have we cut ourselves too short in mapping CTL epitopes? Trends Immunol 2006 27 11 16 10.1016/j.it.2005.11.001 16297661

[B33] Ebert LM Liu YC Clements CS Robson NC Jackson HM Markby JL Dimopoulos N Tan BS Luescher IF Davis ID A long, naturally presented immunodominant epitope from NY-ESO-1 tumor antigen: implications for cancer vaccine design. Cancer Res 2009 69 1046 1054 10.1158/0008-5472.CAN-08-2926 19176376

[B34] Halling-Brown M Shaban R Frampton D Sansom CE Davies M Flower D Duffield M Titball RW Brusic V Moss DS Proteins accessible to immune surveillance show significant T-cell epitope depletion: Implications for vaccine design. Mol Immunol 2009 46 2699 2705 10.1016/j.molimm.2009.05.027 19560824

[B35] Halling-Brown M Sansom CE Davies M Titball RW Moss DS Are bacterial vaccine antigens T-cell epitope depleted? Trends Immunol 2008 29 374 379 10.1016/j.it.2008.06.001 18603471

[B36] Bru C Courcelle E Carrere S Beausse Y Dalmar S Kahn D The ProDom database of protein domain families: more emphasis on 3D. Nucleic Acids Res 2005 33 D212 215 10.1093/nar/gki034 15608179 PMC539988

[B37] Finn RD Mistry J Tate J Coggill P Heger A Pollington JE Gavin OL Gunasekaran P Ceric G Forslund K The Pfam protein families database. Nucleic Acids Res 2010 38 D211 222 10.1093/nar/gkp985 19920124 PMC2808889

[B38] Sigrist CJ Cerutti L de Castro E Langendijk-Genevaux PS Bulliard V Bairoch A Hulo N PROSITE, a protein domain database for functional characterization and annotation. Nucleic Acids Res 2010 38 D161 166 10.1093/nar/gkp885 19858104 PMC2808866

[B39] Radisky DC Stallings-Mann M Hirai Y Bissell MJ Single proteins might have dual but related functions in intracellular and extracellular microenvironments. Nat Rev Mol Cell Biol 2009 10 228 234 10.1038/nrm2633 19190671 PMC2746016

[B40] Emanuelsson O Brunak S von Heijne G Nielsen H Locating proteins in the cell using TargetP, SignalP and related tools. Nat Protoc 2007 2 953 971 10.1038/nprot.2007.131 17446895

[B41] Bendtsen JD Nielsen H von Heijne G Brunak S Improved prediction of signal peptides: SignalP 3.0. J Mol Biol 2004 340 783 795 10.1016/j.jmb.2004.05.028 15223320

[B42] Choo KH Tan TW Ranganathan S A comprehensive assessment of N-terminal signal peptides prediction methods. Bmc Bioinformatics 2009 10 Suppl 15 S2 10.1186/1471-2105-10-S15-S2 19958512 PMC2788353

[B43] Horton P Park KJ Obayashi T Fujita N Harada H Adams-Collier CJ Nakai K WoLF PSORT: protein localization predictor. Nucleic Acids Res 2007 35 W585 587 10.1093/nar/gkm259 17517783 PMC1933216

[B44] Chen Y Yu P Luo J Jiang Y Secreted protein prediction system combining CJ-SPHMM, TMHMM, and PSORT. Mamm Genome 2003 14 859 865 10.1007/s00335-003-2296-6 14724739

[B45] Gardy JL Spencer C Wang K Ester M Tusnady GE Simon I Hua S deFays K Lambert C Nakai K Brinkman FS PSORT-B: Improving protein subcellular localization prediction for Gram-negative bacteria. Nucleic Acids Res 2003 31 3613 3617 10.1093/nar/gkg602 12824378 PMC169008

[B46] Nakai K Horton P PSORT: a program for detecting sorting signals in proteins and predicting their subcellular localization. Trends Biochem Sci 1999 24 34 36 10.1016/S0968-0004(98)01336-X 10087920

[B47] Bulashevska A Eils R Predicting protein subcellular locations using hierarchical ensemble of Bayesian classifiers based on Markov chains. Bmc Bioinformatics 2006 7 298 10.1186/1471-2105-7-298 16774677 PMC1525000

[B48] Chen H Huang N Sun Z SubLoc: a server/client suite for protein subcellular location based on SOAP. Bioinformatics 2006 22 376 377 10.1093/bioinformatics/bti822 16339283

[B49] Shen HB Chou KC Gpos-PLoc: an ensemble classifier for predicting subcellular localization of Gram-positive bacterial proteins. Protein Eng Des Sel 2007 20 39 46 10.1093/protein/gzl053 17244638

[B50] Bendtsen JD Nielsen H Widdick D Palmer T Brunak S Prediction of twin-arginine signal peptides. Bmc Bioinformatics 2005 6 167 10.1186/1471-2105-6-167 15992409 PMC1182353

[B51] Juncker AS Willenbrock H Von Heijne G Brunak S Nielsen H Krogh A Prediction of lipoprotein signal peptides in Gram-negative bacteria. Protein Sci 2003 12 1652 1662 10.1110/ps.0303703 12876315 PMC2323952

[B52] Kall L Krogh A Sonnhammer EL Advantages of combined transmembrane topology and signal peptide prediction--the Phobius web server. Nucleic Acids Res 2007 35 W429 432 10.1093/nar/gkm256 17483518 PMC1933244

[B53] Restrepo-Montoya D Vizcaino C Nino LF Ocampo M Patarroyo ME Patarroyo MA Validating subcellular localization prediction tools with mycobacterial proteins. Bmc Bioinformatics 2009 10 134 10.1186/1471-2105-10-134 19422713 PMC2685389

[B54] Taylor PD Attwood TK Flower DR Toward bacterial protein sub-cellular location prediction: single-class discrimminant models for all gram- and gram+ compartments. Bioinformation 2006 1 276 280 17597907 10.6026/97320630001276 PMC1891713

[B55] Taylor PD Attwood TK Flower DR Multi-class subcellular location prediction for bacterial proteins. Bioinformation 2006 1 260 264 17597904 10.6026/97320630001260 PMC1891703

[B56] Taylor PD Toseland CP Attwood TK Flower DR Alpha helical trans-membrane proteins: Enhanced prediction using a Bayesian approach. Bioinformation 2006 1 234 236 17597896 PMC1891692

[B57] Taylor PD Toseland CP Attwood TK Flower DR Beta barrel trans-membrane proteins: Enhanced prediction using a Bayesian approach. Bioinformation 2006 1 231 233 17597895 PMC1891693

[B58] Taylor PD Toseland CP Attwood TK Flower DR A predictor of membrane class: Discriminating alpha-helical and beta-barrel membrane proteins from non-membranous proteins. Bioinformation 2006 1 208 213 17597890 PMC1891694

[B59] Taylor PD Toseland CP Attwood TK Flower DR TATPred: a Bayesian method for the identification of twin arginine translocation pathway signal sequences. Bioinformation 2006 1 184 187 17597885 10.6026/97320630001184 PMC1891679

[B60] Taylor PD Toseland CP Attwood TK Flower DR LIPPRED: A web server for accurate prediction of lipoprotein signal sequences and cleavage sites. Bioinformation 2006 1 176 179 17597883 10.6026/97320630001176 PMC1891677

[B61] Taylor PD Attwood TK Flower DR Combining algorithms to predict bacterial protein sub-cellular location: Parallel versus concurrent implementations. Bioinformation 2006 1 285 289 17597909 10.6026/97320630001285 PMC1891705

[B62] Guo T Hua S Ji X Sun Z DBSubLoc: database of protein subcellular localization. Nucleic Acids Res 2004 32 D122 124 10.1093/nar/gkh109 14681374 PMC308843

[B63] Scott MS Oomen R Thomas DY Hallett MT Predicting the subcellular localization of viral proteins within a mammalian host cell. Virol J 2006 3 24 10.1186/1743-422X-3-24 16595001 PMC1475561

[B64] Shen HB Chou KC Virus-PLoc: a fusion classifier for predicting the subcellular localization of viral proteins within host and virus-infected cells. Biopolymers 2007 85 233 240 10.1002/bip.20640 17120237

[B65] Schuler MM Nastke MD Stevanovikc S SYFPEITHI: database for searching and T-cell epitope prediction. Methods Mol Biol 2007 409 75 93 full_text 18449993 10.1007/978-1-60327-118-9_5

[B66] Rammensee H Bachmann J Emmerich NP Bachor OA Stevanovic S SYFPEITHI: database for MHC ligands and peptide motifs. Immunogenetics 1999 50 213 219 10.1007/s002510050595 10602881

[B67] Kuiken C Korber B Shafer RW HIV sequence databases. AIDS Rev 2003 5 52 61 12875108 PMC2613779

[B68] Lata S Bhasin M Raghava GP MHCBN 4.0: A database of MHC/TAP binding peptides and T-cell epitopes. BMC Res Notes 2009 2 61 10.1186/1756-0500-2-61 19379493 PMC2679046

[B69] Bhasin M Singh H Raghava GP MHCBN: a comprehensive database of MHC binding and non-binding peptides. Bioinformatics 2003 19 665 666 10.1093/bioinformatics/btg055 12651731

[B70] Reche PA Zhang H Glutting JP Reinherz EL EPIMHC: a curated database of MHC-binding peptides for customized computational vaccinology. Bioinformatics 2005 21 2140 2141 10.1093/bioinformatics/bti269 15657103

[B71] Toseland CP Clayton DJ McSparron H Hemsley SL Blythe MJ Paine K Doytchinova IA Guan P Hattotuwagama CK Flower DR AntiJen: a quantitative immunology database integrating functional, thermodynamic, kinetic, biophysical, and cellular data. Immunome Res 2005 1 4 10.1186/1745-7580-1-4 16305757 PMC1289288

[B72] McSparron H Blythe MJ Zygouri C Doytchinova IA Flower DR JenPep: a novel computational information resource for immunobiology and vaccinology. J Chem Inf Comput Sci 2003 43 1276 1287 12870921 10.1021/ci030461e

[B73] Blythe MJ Doytchinova IA Flower DR JenPep: a database of quantitative functional peptide data for immunology. Bioinformatics 2002 18 434 439 10.1093/bioinformatics/18.3.434 11934742

[B74] Wassenaar TM Gaastra W Bacterial virulence: can we draw the line? FEMS Microbiol Lett 2001 201 1 7 10.1111/j.1574-6968.2001.tb10724.x 11445159

[B75] Yang J Chen L Sun L Yu J Jin Q VFDB 2008 release: an enhanced web-based resource for comparative pathogenomics. Nucleic Acids Res 2008 36 D539 542 10.1093/nar/gkm951 17984080 PMC2238871

[B76] Chen L Yang J Yu J Yao Z Sun L Shen Y Jin Q VFDB: a reference database for bacterial virulence factors. Nucleic Acids Res 2005 33 D325 328 10.1093/nar/gki008 15608208 PMC539962

[B77] Tongchusak S Chaiyaroj SC Veeramani A Koh JLY Brusic V CandiVF - Candida albicans virulence factor database. Int J Pept Res Ther 2005 11 271 277 10.1007/s10989-005-9268-5

[B78] Winnenburg R Baldwin TK Urban M Rawlings C Kohler J Hammond-Kosack KE PHI-base: a new database for pathogen host interactions. Nucleic Acids Res 2006 34 D459 D464 10.1093/nar/gkj047 16381911 PMC1347410

[B79] Ansari HR Flower DR Raghava GPS AntigenDB: an immunoinformatics database of pathogen antigens. Nucleic Acids Res 2010 38 D847 D853 10.1093/nar/gkp830 19820110 PMC2808902

[B80] Flower DR The lipocalin protein family: Structure and function. Biochem J 1996 318 1 14 8761444 10.1042/bj3180001 PMC1217580

[B81] Flower DR North ACT Attwood TK Structure and Sequence Relationships in the Lipocalins and Related Proteins. Protein Sci 1993 2 753 761 10.1002/pro.5560020507 7684291 PMC2142497

[B82] Flower DR North AC Sansom CE The lipocalin protein family: structural and sequence overview. Biochim Biophys Acta 2000 1482 9 24 11058743 10.1016/s0167-4838(00)00148-5

[B83] Kanduc D Epitopic peptides with low similarity to the host proteome: towards biological therapies without side effects. Expert Opin Biol Ther 2009 9 45 53 10.1517/14712590802614041 19063692

[B84] Kanduc D Peptimmunology: immunogenic peptides and sequence redundancy. Curr Drug Discov Technol 2005 2 239 244 10.2174/157016305775202946 16475920

[B85] Singh NJ Schwartz RH Primer: mechanisms of immunologic tolerance. Nat Clin Pract Rheumatol 2006 2 44 52 10.1038/ncprheum0049 16932651

[B86] Miao CH Recent advances in immune modulation. Curr Gene Ther 2007 7 391 402 10.2174/156652307782151524 17979685

[B87] Barron L Knoechel B Lohr J Abbas AK Cutting edge: contributions of apoptosis and anergy to systemic T cell tolerance. J Immunol 2008 180 2762 2766 18292495 10.4049/jimmunol.180.5.2762

[B88] Yoon SH Hur CG Kang HY Kim YH Oh TK Kim JF A computational approach for identifying pathogenicity islands in prokaryotic genomes. Bmc Bioinformatics 2005 6 184 10.1186/1471-2105-6-184 16033657 PMC1188055

[B89] Guy L Identification and characterization of pathogenicity and other genomic islands using base composition analyses. Future Microbiol 2006 1 309 316 10.2217/17460913.1.3.309 17661643

[B90] Wolff K Stern A Identification and characterization of specific sequences encoding pathogenicity associated proteins in the genome of commensal Neisseria species. FEMS Microbiol Lett 1995 125 255 263 10.1111/j.1574-6968.1995.tb07366.x 7875573

[B91] Wang G Zhou F Olman V Li F Xu Y Prediction of pathogenicity islands in enterohemorrhagic Escherichia coli O157:H7 using genomic barcodes. Febs Lett 2010 584 194 198 10.1016/j.febslet.2009.11.067 19941858

[B92] Hackenberg M Previti C Luque-Escamilla PL Carpena P Martinez-Aroza J Oliver JL CpGcluster: a distance-based algorithm for CpG-island detection. Bmc Bioinformatics 2006 7 446 10.1186/1471-2105-7-446 17038168 PMC1617122

[B93] Sujuan Y Asaithambi A Liu Y CpGIF: an algorithm for the identification of CpG islands. Bioinformation 2008 2 335 338 18685720 10.6026/97320630002335 PMC2478732

[B94] Hutter B Paulsen M Helms V Identifying CpG Islands by Different Computational Techniques. OMICS 2009 19196100 10.1089/omi.2008.0046

[B95] Su J Zhang Y Lv J Liu H Tang X Wang F Qi Y Feng Y Li X CpG_MI: a novel approach for identifying functional CpG islands in mammalian genomes. Nucleic Acids Res 2010 38 e6 10.1093/nar/gkp882 19854943 PMC2800233

[B96] Flower DR North AC Attwood TK Structure and sequence relationships in the lipocalins and related proteins. Protein Sci 1993 2 753 761 10.1002/pro.5560020507 7684291 PMC2142497

[B97] Flower DR Structural Relationship of Streptavidin to the Calycin Protein Superfamily. Febs Lett 1993 333 99 102 10.1016/0014-5793(93)80382-5 8224179

[B98] Doytchinova IA Flower DR VaxiJen: a server for prediction of protective antigens, tumour antigens and subunit vaccines. Bmc Bioinformatics 2007 8 10.1186/1471-2105-8-4 17207271 PMC1780059

[B99] Doytchinova IA Flower DR Identifying candidate subunit vaccines using an alignment-independent method based on principal amino acid properties. Vaccine 2007 25 856 866 10.1016/j.vaccine.2006.09.032 17045707

[B100] Wold S Jonsson J Sjostrom M Sandberg M Rannar S DNA and Peptide Sequences and Chemical Processes Multivariately Modeled by Principal Component Analysis and Partial Least-Squares Projections to Latent Structures. Anal Chim Acta 1993 277 239 253 10.1016/0003-2670(93)80437-P

[B101] Wold S Eriksson L Hellberg S Jonsson J Sjostrom M Skagerberg B Wikstrom C Principal Property-Values for 6 Nonnatural Amino-Acids and Their Application to a Structure Activity Relationship for Oxytocin Peptide Analogs. Can J Chem 1987 65 1814 1820 10.1139/v87-305

[B102] Dimitrov I Garnev P Flower DR Doytchinova I Peptide binding to the HLA-DRB1 supertype: a proteochemometrics analysis. Eur J Med Chem 2010 45 236 243 10.1016/j.ejmech.2009.09.049 19896246

[B103] Kontijevskis A Petrovska R Yahorava S Komorowski J Wikberg JE Proteochemometrics mapping of the interaction space for retroviral proteases and their substrates. Bioorg Med Chem 2009 17 5229 5237 10.1016/j.bmc.2009.05.045 19539482

[B104] Prusis P Lapins M Yahorava S Petrovska R Niyomrattanakit P Katzenmeier G Wikberg JE Proteochemometrics analysis of substrate interactions with dengue virus NS3 proteases. Bioorg Med Chem 2008 16 9369 9377 10.1016/j.bmc.2008.08.081 18824362

[B105] Strombergsson H Kryshtafovych A Prusis P Fidelis K Wikberg JE Komorowski J Hvidsten TR Generalized modeling of enzyme-ligand interactions using proteochemometrics and local protein substructures. Proteins 2006 65 568 579 10.1002/prot.21163 16948162

[B106] Strombergsson H Prusis P Midelfart H Lapinsh M Wikberg JE Komorowski J Rough set-based proteochemometrics modeling of G-protein-coupled receptor-ligand interactions. Proteins 2006 63 24 34 10.1002/prot.20777 16435365

[B107] Lapinsh M Prusis P Uhlen S Wikberg JE Improved approach for proteochemometrics modeling: application to organic compound--amine G protein-coupled receptor interactions. Bioinformatics 2005 21 4289 4296 10.1093/bioinformatics/bti703 16204343

[B108] Wikberg JE Mutulis F Mutule I Veiksina S Lapinsh M Petrovska R Prusis P Melanocortin receptors: ligands and proteochemometrics modeling. Ann N Y Acad Sci 2003 994 21 26 10.1111/j.1749-6632.2003.tb03158.x 12851294

[B109] Lapinsh M Prusis P Lundstedt T Wikberg JE Proteochemometrics modeling of the interaction of amine G-protein coupled receptors with a diverse set of ligands. Mol Pharmacol 2002 61 1465 1475 10.1124/mol.61.6.1465 12021408

[B110] Hellberg S Sjostrom M Skagerberg B Wold S Peptide Quantitative Structure-Activity-Relationships, a Multivariate Approach. J Med Chem 1987 30 1126 1135 10.1021/jm00390a003 3599020

[B111] Jonsson J Eriksson L Hellberg S Sjostrom M Wold S Multivariate Parametrization of 55 Coded and Non-Coded Amino-Acids. Quant Struct-Act Rel 1989 8 204 209 10.1002/qsar.19890080303

[B112] Sandberg M Eriksson L Jonsson J Sjostrom M Wold S New chemical descriptors relevant for the design of biologically active peptides. A multivariate characterization of 87 amino acids. J Med Chem 1998 41 2481 2491 10.1021/jm9700575 9651153

[B113] Doytchinova IA Flower DR Bioinformatic Approach for Identifying Parasite and Fungal Candidate Subunit Vaccines. The Open Vaccine Journal 2008 1 4 10.2174/1875035400801010022

[B114] Vivona S Bernante F Filippini F NERVE: new enhanced reverse vaccinology environment. BMC Biotechnol 2006 6 35 10.1186/1472-6750-6-35 16848907 PMC1570458

[B115] Harish N Gupta R Agarwal P Scaria V Pillai B DyNAVacS: an integrative tool for optimized DNA vaccine design. Nucleic Acids Res 2006 34 W264 266 10.1093/nar/gkl242 16845007 PMC1538838

[B116] Fell DA Enzymes, metabolites and fluxes. J Exp Bot 2005 56 267 272 10.1093/jxb/eri011 15545297

